# Exploring the effects of music-supported cultural adaptation activities on international students’ sociocultural adaptation and wellbeing: a pilot study

**DOI:** 10.3389/fpsyg.2026.1828413

**Published:** 2026-06-05

**Authors:** Erkan Sulun, Ozbil Kurtulmus, Hakki Cengiz Eren

**Affiliations:** 1 Ministry of Education, Nicosia, Cyprus; 2 Music Teaching Department, Near East University, Nicosia, Cyprus

**Keywords:** international students, intervention, music, sociocultural adaptation, wellbeing

## Abstract

International student mobility has increased in recent years with the internationalization of higher education. However, this process may lead to acculturative stress, creating challenges for students’ sociocultural adaptation and wellbeing. Music has been proposed as a potential tool to support both sociocultural adaptation and wellbeing. This pilot study examined the preliminary effects of a five-week music-supported cultural adaptation program on the sociocultural adaptation and wellbeing of international students in Northern Cyprus. A one-group pretest–posttest design was employed with 10 participants. Quantitative data were assessed using the SCAS-R, WHO-5, and the MHC-SF, while participant journals were used to provide supplementary reflective context for the quantitative findings. Quantitative data were analyzed using SPSS, and differences between pretest–posttest scores were examined using the Wilcoxon signed-rank test. Positive trends were observed in overall sociocultural adaptation (*p* = 0.009), particularly in the interpersonal communication (*p* = 0.008) and ecological adaptation (*p* = 0.014) subscales of the SCAS-R. Positive trends were also observed in mental wellbeing (*p* = 0.008) on the WHO-5 and in the emotional wellbeing (*p* = 0.007) and social wellbeing (*p* = 0.006) dimensions of MHC-SF. However, given the small sample size, absence of a control group, and single-group design, these findings should be interpreted with considerable caution and cannot be attributed exclusively to the music-supported activities. These findings suggest that music-supported cultural adaptation activities may warrant further investigation, and future research with larger samples and controlled designs is needed to evaluate their effectiveness.

## 1 Introduction

International student mobility has increased in recent years as higher education becomes internationalized and cross-border education continues to grow (Altbach and Knight, 2007). As a result, a growing number of students pursue their higher education abroad for academic development, intercultural competence, personal growth and better career opportunities (Dwyer, 2004). However, adapting to a foreign country can be challenging and stressful for international students (Rienties and Tempelaar, 2013). This often results in acculturative stress as international students face new challenges such as language barriers, academic challenges, social integration difficulties and unfamiliar cultural norms, all of which may affect their adaptation and wellbeing (Koo et al., 2021).

Research has shown that acculturative stress has a negative effect on international students’ wellbeing, social engagement, and academic performance (DeLancey, 2021). High levels of acculturative stress are strongly associated with lower life satisfaction, increased anxiety, decreased psychological wellbeing, and general adjustment difficulties (Yoon et al., 2008; Mesidor and Sly, 2016). Su et al. (2021) found that Chinese students studying in the United States reported significantly lower quality of life and higher stress due to cultural differences. Ali et al. (2024) found that international students in Egypt faced difficulties in communication and academic engagement due to cultural stress. Jarrar and Nweke (2025) found that Nigerian students in Cyprus were negatively affected by acculturative stress, which hindered their adjustment to university life.

Acculturation Theory provides a fundamental perspective for understanding how individuals experience and manage cultural transitions across sociocultural boundaries. According to Berry (2005), cross-cultural adaptation is a multidimensional and complex process involving both cultural and psychological changes. These changes result from interactions between culturally diverse groups. Cross cultural adaptation can be categorized into two dimensions: sociocultural adaptation and psychological adaptation (Ward and Kennedy, 1999). This theoretical framework has transformed the acculturation concept into a functional field of research, allowing researchers to systematically examine the multifaceted nature of acculturation processes. Among these two dimensions, sociocultural adaptation is defined as the process of an individual adapting to a host society (Ward and Fischer, 2008). On the other hand, psychological adaptation has been conceptualized through the affective dimension as the sense of happiness and satisfaction (Ward and Kennedy, 1999).

In developing education regions such as Northern Cyprus international students constitute a significant portion of the higher education population. According to the Ministry of National Education and Culture, recent data shows that there are 103,000 students enrolled at 23 universities in Northern Cyprus and approximately 42,000 of these students are international students (MEB, 2025). Students from Africa, Asia, and the Middle East study together in a diverse environment, cultural integration occurs through different forms of interaction, such as participating in local traditions and experiencing the host country’s music. These activities highlight how individuals slowly become more familiar with and comfortable in the host society, aligning their attitudes and social behaviors with local norms over time (Ward and Kennedy, 1999). According to Acculturation Theory, interacting with the host society’s cultural and musical elements can strengthen social bonds and increase feelings of belonging (Crooke et al., 2024).

Participating in the host culture and music can strengthen social bonds, reduce perceived cultural differences, and encourage shared experiences that support interpersonal communication (Chen et al., 2020). International students face challenges related to language, culture, and identity. However, this type of interaction allows them to explore a new cultural environment while preserving aspects of their original identity (Bond, 2022). Students who effectively manage the acculturation process tend to develop a stronger sense of community and shared responsibility, which can support the sustainability of multicultural learning environments (Makarova and Sidler, 2023). Given the strong link between acculturation difficulties and wellbeing, music has been proposed as an effective tool that can support both sociocultural adaptation and wellbeing processes. Music has long played such a role in human societies and has been used throughout history in rituals, social events, and cultural traditions. Research shows that music has a powerful effect in reducing stress and anxiety, increasing positive effects, and enhancing psychological wellbeing (Fancourt and Finn, 2019). Music brings people together and makes social interaction easier (DeNora, 2000).

Studies show that community music activities, especially making music together, help people from different cultural backgrounds communicate more easily, reduce prejudices, and strengthen their sense of connection and belonging (Higgins, 2012; Joseph and Southcott, 2014). Many of these academic, social, and cultural difficulties suggest that international students often need environments that help them feel connected, reduce stress, and ease their transition into a new society (Smith and Khawaja, 2011; Zhang and Goodson, 2011). Although universities offer different types of support, these services mainly focus on academic or administrative matters, and students’ social and emotional needs often remain in the background (Sawir et al., 2008; Han et al., 2017). Despite these findings, several gaps remain. First, most studies have focused on international students in English-speaking countries such as Australia, the United Kingdom, and the United States (Su et al., 2021; Ali et al., 2024). There is a lack of studies on international students in Northern Cyprus despite the region hosting a large international student population. Second, there are limited studies employing music-supported cultural adaptation activities to examine international students’ sociocultural adaptation and wellbeing in higher education. Building on the reviewed literature, the following hypotheses are proposed:

*H1*. Music-supported cultural adaptation activities are expected to be associated with improvements in overall sociocultural adaptation (SCAS-R total score).

*H2*. Music-supported cultural adaptation activities are expected to be associated with improvements in mental wellbeing (WHO-5 score).

*H3*. Music-supported cultural adaptation activities are expected to be associated with improvements in mental health (MHC-SF total score).

## 2 Methods

This study evaluated the effects of a 5-week, music-supported cultural adaptation intervention on international students’ sociocultural adaptation and wellbeing. Given the exploratory and pilot nature of the intervention, a one-group pretest–posttest design was employed to assess feasibility and preliminary outcome trends (Julious, 2005). Data were collected at two time points: baseline (pre-intervention) and after 5 weeks (post-intervention). Quantitative data were measured using the Sociocultural Adaptation Scale-Revised (SCAS-R), WellBeing Index (WHO-5), and Mental Health Continuum-Short Form (MHC-SF). In addition, reflective journal data were collected through participants’ journals, which were completed after each session to provide supplementary contextual insight into the intervention process.

### 2.1 Measurement tools

#### 2.1.1 Demographic information form

In addition to the quantitative and reflective journal data, demographic information was collected using Google Forms. Participants provided basic information including age, gender, nationality, university, work status, years in Northern Cyprus, and level of education.

#### 2.1.2 Sociocultural adaptation scale-revised (SCAS-R)

The Sociocultural Adaptation Scale-Revised (SCAS-R) is a widely used instrument for assessing international students’ sociocultural adaptation across multiple domains (Wilson, 2013). The SCAS-R evaluates the extent of difficulties experienced in adapting to a new cultural environment using a 5-point Likert scale (1 = not competent at all, 5 = extremely competent). The instrument consists of 21 items grouped under five subscales: interpersonal communication (e.g., making friends, understanding jokes), academic/work adjustment (e.g., understanding lectures, interacting with teachers), social participation and community involvement (e.g., joining social activities, meeting local people), ecological adaptation (e.g., transportation, shopping, local food), and language proficiency (e.g., using the local language in daily interactions).

#### 2.1.3 WellBeing Index (WHO-5)

The WHO-5 WellBeing Index was used for the evaluation of participants’ mental wellbeing (Topp et al., 2015). WHO-5 consists of five positively worded statements on a 6-point Likert scale. Scores range from 0 to 25 and are multiplied by four to obtain a final score between 0 and 100, with higher scores indicating greater wellbeing. A score below 13 suggests poor wellbeing (Brähler et al., 2007).

#### 2.1.4 Mental health continuum short form (MHC-SF)

The MHC-SF is a self-report scale that measures emotional, social, and psychological wellbeing characteristics representing the mental health continuum. The scale consists of 14 items and three subscales: items 1, 2, and 3 address emotional wellbeing; items 4, 5, 6, 7, and 8 address social wellbeing; and items 9, 10, 11, 12, 13, and 14 address psychological wellbeing. The scale is formatted on a 6-point Likert scale (0 = Never, 5 = Every day). The possible score on the scale ranges from 0 to 70. There are no reverse-scored items. A total score for mental health is obtained by summing the 14 items on the scale. The emotional, social, and psychological wellbeing subscales can also be scored separately. High scores on each subscale indicate high wellbeing in that area. Those who select “almost every day” or “every day” on one of the three statements in the emotional wellbeing dimension, and “almost every day” or “every day” on six of the 11 statements in the psychological and social wellbeing dimensions, are defined as “flourishing.” Those who marked “never” or “once or twice” on one of the three items in the emotional wellbeing dimension, and “never” or “once or twice” on six of the 11 items in the psychological and social wellbeing dimensions, are defined as “languishing.” Those who remain outside these limits are considered to be in moderate mental health (Keyes et al., 2008).

#### 2.1.5 Participant journal

Participant journals are widely used in intervention research as a data collection tool, as they allow individuals to express their experiences, feelings, and thoughts throughout a research process from their own perspective. Particularly in intervention-based studies, they help researchers gain an inside view by allowing for an in-depth examination of the effects of the intervention on participants (Alaszewski, 2006). Participant journals serve as a complementary reflective data source that supports and contextualizes the findings obtained in the quantitative dimension of the research (Creswell, 2017). Structured participant journals facilitate participants’ reflection on specific themes and enable more systematic monitoring of changes that occur during the process (Jacelon and Imperio, 2005). In the present study, journal entries were not subjected to formal qualitative analysis; rather, selected quotations are presented in the discussion section to provide supplementary illustrative context for the quantitative findings.

### 2.2 Data analysis

All quantitative data obtained from the scales were analyzed using SPSS version 26. Demographic information was summarized using frequency tables. To examine the differences between students’ pretest–posttest scores on the SCAS-R, WHO-5, and MHC-SF, the Wilcoxon signed-rank test was employed as the appropriate non-parametric alternative to the paired samples *t*-test, given the small sample size (*n* = 10) and the ordinal nature of the Likert-scale data, which precluded the assumptions required for parametric testing (Field, 2013). In addition, reflective journal entries are presented in the discussion section through direct quotations to provide supplementary contextual interpretation of the changes observed in the quantitative results. Participants were assigned codes from P1 to P10 to ensure anonymity. The journals were completed using guided questions that asked participants to reflect on their experiences during the music-supported cultural adaptation activities.

### 2.3 Participants

The study was conducted with 10 participants enrolled at Near East University and Cyprus International University during the spring semester of the 2024–2025 academic year. The small sample size was due to the limited availability of students who could regularly attend the weekly sessions. Reaching a large sample was not feasible at this stage, as small sample sizes are typical and acceptable in pilot studies (Julious, 2005; Hertzog, 2008). Inclusion criteria required that participants (a) were international students, (b) had adequate English proficiency, and (c) were able to attend 5 weekly sessions. No musical experience was required. Recruitment occurred through university email lists, international student offices, student clubs, and social media postings. All sessions lasted 90 min and were designed in accordance with the subscales of the SCAS-R. Participation was voluntary, and informed consent was obtained from all students. They were assured of confidentiality with no personal information recorded and informed that their responses would be exclusively utilized for research purposes. The activities were conducted between 9 May 2025 and 6 June 2025. Participant attendance was consistent throughout the intervention; all 10 participants completed the full 5-week program and attended all five scheduled sessions without exception. The research was authorized by the Near East University’s Research Ethics Committee (approval number: YDÜ/EB/2024/1100).

### 2.4 Weekly plan of activities

To support intervention fidelity, all sessions followed a predefined activity structure and were facilitated by the same researcher throughout the 5-week intervention period. The sessions were facilitated by the second author (OK), who holds a bachelor’s degree in music teaching and a master’s degree in music education, ensuring both musical competence and pedagogical experience throughout the intervention. Although no formal fidelity checklist or independent fidelity assessment was implemented, consistency was maintained through a structured session format and single-facilitator delivery. This represents a limitation of the present study and future research should consider incorporating formal fidelity monitoring procedures. All five sessions were held in the same music studio throughout the intervention.

#### 2.4.1 Week 1: Interpersonal communication

The first session was designed to encourage interpersonal communication and to reduce social barriers among the participants. The session took place in a music studio that was arranged to create a relaxed and informal atmosphere, with seating organized in a circle to facilitate open conversation and social interaction, along with sufficient space and audio equipment for all participants. At the beginning of the session, students were introduced to the Cypriot coffee-sharing and conversation culture. This tradition was presented as a social practice that brings people together and encourages informal conversation. During this activity, students briefly introduced themselves and engaged in conversations with fellow participants. As part of the session, students listened to selected Cypriot traditional folk songs. After that, each participant was asked to share a song from their own country and briefly explain its cultural meaning or personal importance to the group. Afterward, a short group reflection was held in which participants discussed similarities and differences between the musical traditions that were shared. Overall, these activities aimed to encourage self-expression, active listening, and intercultural exchange while helping participants develop initial interpersonal connections. By providing a relaxed and supportive environment, the session also aimed to reduce social discomfort and increase participants’ confidence in interacting with others in the group.

#### 2.4.2 Week 2: Academic/work performance

The second session focused on helping students become familiar with the academic environment in the Northern Cyprus. The session began with a brief explanation of common expectations at local universities, such as how students participate in class, communicate with lecturers, and approach assignments. After this introduction, students took part in a rhythm-based group activity. Each group created a basic rhythm pattern and performed it using body percussion. This activity encouraged participants to listen to each other and coordinate their actions, while shared responsibility within the group helped highlight the importance of teamwork (Hove and Risen, 2009; Kirschner and Tomasello, 2010). The session ended with a short discussion in which students compared the academic practices of their home countries with those in the Northern Cyprus and reflected on how the musical activity related to challenges such as managing time or working within a group.

#### 2.4.3 Week 3: Personal interests and community involvement

The third session focused on helping students establish a connection with the community through music and folk dance. The session started with a short introduction to Cypriot folk-dance traditions. Students were presented with physical examples of traditional costumes displayed in the studio. Traditional music was played live during the session, and students practiced simple dance steps in accompaniment with the music, as group movement has been associated with social bonding (Tarr et al., 2014). Later, students moved into small groups and talked about their hobbies and cultural habits from their home countries. Each group prepared a small sharing activity using music and dance that reflected their culture. The session aimed to help students feel closer to the host community by linking their interests with local cultural life. It also encouraged social interaction and helped students feel more comfortable engaging with each other.

#### 2.4.4 Week 4: Ecological adaptation

The fourth session focused on helping students become more familiar with the everyday environment of Northern Cyprus. The session began with an introduction to local historical places in the old town of Nicosia. In combination with a cultural walk, students were able to see local people and feel the atmosphere of the town, listening to ambient sounds, including street music, local melodies, and natural soundscapes. During the walk, participants also sampled a selection of local foods, allowing them to experience how taste, music, and place interact together in everyday life. After the activity, students discussed what they noticed and shared impressions of similar places in their home countries and reflected on how becoming familiar with the environment could support adaptation, as environmental familiarity has been linked to adjustment (Brown and Perkins, 1992). The session aimed to help students feel more comfortable in their surroundings by linking cultural experience, sensory awareness, and ecological understanding.

#### 2.4.5 Week 5: Language proficiency

The fifth session focused on helping students improve their knowledge and use of the host language with the help of music for everyday expressions and cultural interactions. The session began with an introduction to commonly used Turkish words, including basic greetings, common expressions, and phrases used frequently in daily communications. Students then took part in learning a Turkish song to practice pronunciation through repetition and rhythm. This helped students feel more comfortable with pronunciation. The aim was to lower anxiety caused by language barriers and help them feel more confident. These activities were selected based on evidence that participatory music-making enhances social identity, reduces anxiety, and promotes intercultural understanding (DeNora, 2000; Higgins, 2012; Joseph and Southcott, 2014).

## 3 Results

### 3.1 Demographic information form

Table 1 shows the Demographic Information Form of the participants. The sample consisted of two participants aged 18–21, five aged 22–25, and two aged 26 or older. The gender distribution was equal, with five female and five male participants. Participants represented diverse national backgrounds, including two students from Congo, two from Nigeria, two from Iran, and one each from Uganda, Cameroon, Botswana, and Mali. Regarding institutional enrollment, six students were registered at Near East University and four at Cyprus International University. In terms of employment, seven participants reported being unemployed and three reported being employed. Regarding length of residence in the TRNC, four participants had been residing for 4–6 years, three for less than 1 year, and three for 1–3 years. In terms of academic level, the sample comprised five undergraduate, four master’s, and one PhD student.

**Table 1:** Distribution of students according to demographic information form.

Variable	Freq (f)	Percent (%)
Age group		
18–21	3	30,00
22–25	5	50,00
26 and older	2	20,00
Gender		
Female	5	50,00
Male	5	50,00
Nationality		
Congolese	2	20,00
Nigerian	2	20,00
Iranian	2	20,00
Ugandan	1	10,00
Cameroonian	1	10,00
Botswanan	1	10,00
Malian	1	10,00
University		
Near East University	6	60,00
Cyprus International University	4	40,00
Work status		
Yes	3	30,00
No	7	70,00
Years in Northern Cyprus		
Less than 1 year	3	30,00
1–3 years	3	30,00
4–6 years	4	40,00
Education level		
Bachelor’s	5	50,00
Master’s	4	40,00
PhD	1	10,00
Total	10	100,00

### 3.2 Sociocultural adaptation scale revised (SCAS-R) pre–post results

Table 2 presents the results of students’ pretest–posttest scores on the Sociocultural Adaptation Scale Revised (SCAS-R). A statistically significant increase was observed in Interpersonal Communication scores, with the median rising from 22.00 at pretest to 23.50 at posttest (*Z* = −2.64, *p* = 0.008, *r* = 0.83). Ecological Adaptation scores also demonstrated significant improvement, increasing from a median of 14.00 to 15.00 (*Z* = −2.449, *p* = 0.014, *r* = 0.77). In contrast, no statistically significant differences were found for Academic/Work Performance (*Z* = −0.577, *p* = 0.564, *r* = 0.18) or Personal Interests and Community Involvement (*Z* = −1.633, *p* = 0.102, *r* = 0.52). Although Language Proficiency scores increased from a median of 4.50 to 5.00, the change did not reach statistical significance (*Z* = −1.89, *p* = 0.059, *r* = 0.60). Overall, the total SCAS-R score increased significantly from a pretest median of 66.50 to a posttest median of 70.50 (*Z* = −2.625, *p* = 0.009, *r* = 0.83).

**Table 2:** Comparison of students’ pretest–posttest scores on the sociocultural adaptation scale revised (SCAS-R).

Variable	*n*	Pre test			Post test			*Z*	*p*	*r*
		$\bar{x}$	*s*	*M*	$\bar{x}$	*s*	*M*			
Interpersonal communication	10	22.20	5.71	22.00	23.20	5.85	23.50	−2.64	0.008^*^	0.83
Academic/work performance	10	13.60	3.41	14.50	13.70	3.59	15.00	−0.577	0.564	0.18
Personal interests and community involvement	10	11.80	3.49	13.00	12.20	3.55	13.50	−1.633	0.102	0.52
Ecological adaptation	10	13.10	4.15	14.00	13.70	3.95	15.00	−2.449	0.014^*^	0.77
Language proficiency	10	4.50	2.32	4.50	5.00	2.36	5.00	−1.89	0.059	0.60
Revised sociocultural adaptation scale (SCAS-R)	10	65.20	12.80	66.50	67.80	13.42	70.50	−2.625	0.009^*^	0.83

^*^p < 0.05.

### 3.3 WellBeing Index (WHO-5) pre–post results

Table 3 presents the results of students’ pretest–posttest scores on the WellBeing Index (WHO-5). A statistically significant improvement in mental wellbeing was observed from pretest (Mdn = 11.50) to posttest (Mdn = 13.00), *Z* = −2.65, *p* = 0.008, *r* = 0.84. Mean scores also increased from 11.90 (SD = 5.63) at pretest to 13.40 (SD = 5.63) at posttest.

**Table 3:** Comparison of students’ pretest–posttest scores on WellBeing Index (WHO-5).

Variable	*n*	Pre test			Post test			*Z*	*p*	*r*
		$\bar{x}$	*s*	*M*	$\bar{x}$	*s*	*M*			
WHO-5 WellBeing	10	11.90	5.63	11.50	13.40	5.63	13.00	−2.65	0.008*	0.84

^*^p < 0.05.

### 3.4 Mental health continuum-short form (MHC-SF) pre–post results

Table 4 presents the results of students’ pre- and posttest scores on the Mental Health Continuum–Short Form (MHC-SF). A statistically significant increase was observed in Emotional WellBeing, with median scores rising from 7.00 at pretest to 8.00 at posttest (*Z* = −2.76, *p* = 0.007, *r* = 0.87). Mean scores also increased from 6.80 (SD = 2.70) to 8.00 (SD = 2.60). Similarly, Social WellBeing demonstrated a statistically significant improvement (*Z* = −2.69, *p* = 0.006, *r* = 0.85), with median scores increasing from 10.50 to 11.50 and mean scores rising from 9.70 (SD = 4.40) to 11.20 (SD = 4.10). In contrast, although psychological wellbeing showed a slight increase, with median scores rising from 14.50 to 15.00 and mean scores increasing from 14.70 (SD = 4.40) to 15.10 (SD = 4.30), this change did not reach statistical significance (*Z* = −1.94, *p* = 0.051, *r* = 0.61). Finally, the overall MHC-SF total score demonstrated a statistically significant increase from a pretest median of 45.50 to a posttest median of 48.50 (*Z* = −2.49, *p* = 0.013, *r* = 0.79). Mean scores also increased from 46.80 (SD = 12.00) to 49.90 (SD = 11.30).

**Table 4:** Comparisons of pretest–posttest scores on the Mental Health Continuum–Short Form (MHC-SF).

Variable	*n*	Pre test			Post test			*Z*	*p*	*r*
		$\bar{x}$	*s*	*M*	$\bar{x}$	*s*	*M*			
Emotional WellBeing	10	6.80	2.70	7.00	8.00	2.60	8.00	−2.76	0.007^*^	0.87
Social WellBeing	10	9.70	4.40	10.50	11.20	4.10	11.50	−2.69	0.006^*^	0.85
Psychological wellbeing	10	14.70	4.40	14.50	15.10	4.30	15.00	−1.94	0.051	0.61
MHC-SF total score	10	46.80	12.00	45.50	49.90	11.30	48.50	−2.49	0.013^*^	0.79

^*^p < 0.05.

## 4 Discussion

This pilot study examined the potential effects of music-supported cultural adaptation activities on international students’ sociocultural adaptation and wellbeing. Given the pilot nature of this study, a small sample of 10 participants, the absence of a control group, and a 5-week intervention period, causal claims cannot be made, and the findings should be interpreted within these design boundaries. That said, the overall pattern of findings pointed in theoretically meaningful directions and suggest that music-supported cultural activities may constitute a promising avenue for supporting international student adaptation.

### 4.1 Sociocultural adaptation (H1)

When examining the subscales of the SCAS-R, the findings indicate a meaningful improvement in interpersonal communication (*Z* = −2.64, *p* = 0.008, *r* = 0.83). This pattern is consistent with theoretical accounts of music’s social function: group music-making requires mutual attention, synchronization, and cooperation, and these processes have been proposed to act as catalysts for social closeness (Livesey et al., 2012; Glew et al., 2021). For international students, such relational contexts have been associated with reductions in social isolation and cultural alienation (Smith and Khawaja, 2011). The reflective journal entries align with this pattern. Participant 3 described growing to feel more included in the group over the course of the activities, and Participant 1 expressed a developing sense that cultural adaptation need not mean leaving one’s own cultural identity behind. These reflections suggest that music-supported activities may create a safe space where students can express themselves within the host culture without feeling pressured to assimilate. This is consistent with Higgins (2012) argument that music in cross-cultural contexts facilitates communication by transcending some of the limitations of verbal language. The findings also indicate improvement in the ecological adaptation subscale (*Z* = −2.45, *p* = 0.014, *r* = 0.77). This pattern suggests that engagement with local cultural and physical environments through music-based activities contributed to increased environmental familiarity and a growing sense of belonging in the new setting (Berry, 1997). The journal reflections support this interpretation. Participant 2 described feeling less like a stranger after walking through the historic city, and Participant 7 noted that the sounds of the street and local music made the city feel more alive and familiar. Participant 10’s account of shifting from moving only between home and university to noticing the places, sounds, and people around them captures a qualitative shift that is consistent with the quantitative improvement observed on this subscale. Taken together, these findings align with Ward and Kennedy's (1999) characterisation of ecological adaptation as encompassing not only physical navigation but also a felt sense of participation in the rhythms of daily life. No statistically significant change was found in the academic and work performance subscale (*Z* = −0.577, *p* = 0.564, *r* = 0.18). This is consistent with the understanding that academic adjustment is shaped by structural factors well beyond the reach of a brief cultural short-term intervention including language proficiency, pedagogical approaches, and institutional context and that meaningful cognitive and emotional shifts in this domain may not be captured by performance measures over short timeframes (Sawir et al., 2008).

Participant 5 reported perceiving no change in academic outcomes. Notably, however, Participant 8 described a shift in perspective coming to see the challenges they faced as partly systemic rather than purely individual. This kind of cognitive reappraisal, while not reflected in the quantitative subscale, may represent a form of adaptation that is meaningful in its own right and that warrants attention in future research. Similarly, no significant difference was observed in the personal interests and community involvement subscale (*Z* = −1.633, *p* = 0.102, *r* = 0.52). The development of new community ties and personal interests is generally understood to unfold over extended periods through the gradual building of social networks (Smith and Khawaja, 2011), and a 5-week program is unlikely to produce detectable change in this domain. Participant 9’s reflection expressing greater openness to local events but uncertainty about where to begin captures this transitional state and suggests that the program may have planted early motivational seeds that could develop further with sustained support. The language proficiency subscale showed an upward trend that approached but did not reach statistical significance (*Z* = −1.897, *p* = 0.059, *r* = 0.60). This pattern indicates that a brief intervention is unlikely to produce robust measurable language gains, which is consistent with the understanding that language development requires regular, sustained practice over time. Nevertheless, the rhythm-based and singing activities appear to have created conditions that reduced language anxiety and increased participants’ willingness to engage with the host language (MacIntyre, 1999). Participant 3’s account of feeling less afraid of making mistakes when singing in Turkish, and Participant 8’s report of increased confidence following rhythmic repetition, suggest that music may offer a low-threat context for language engagement a hypothesis that warrants further investigation in future research. Taken together, these findings are consistent with Acculturation Theory’s emphasis on the importance of active, non-pressured interaction with the host culture in the adaptation process (Berry, 1997). The pattern of results suggests that music-supported cultural activities may be particularly well-suited to facilitating interpersonal connection and environmental belonging, while the absence of significant effects in other subscales reflects the complexity of adaptation processes and the inherent constraints of a short intervention rather than an absence of potential.

### 4.2 General wellbeing (H2)

The improvements observed in WHO-5 scores (*Z* = −2.65, *p* = 0.008, *r* = 0.84) indicate that participation in the activities was associated with enhanced general wellbeing, consistent with H2. This pattern is consistent with a substantial body of research linking group music-making to mood regulation (Fancourt and Finn, 2019; Tarr et al., 2014). Interpersonal synchrony during music production has been associated with neurobiological mechanisms including oxytocin and endorphin activity that support positive affect (Good et al., 2021), and music listening has been linked to reductions in stress-related physiological responses and improvements in mood (Linnemann et al., 2015). The journal reflections support this interpretation: Participant 3 described feeling relaxed after sessions, as though the stress of the day had lifted, and Participant 1 noted that sessions consistently improved their mood and sense of positivity.

### 4.3 Mental health (H3)

The findings related to mental health are consistent with H3. Significant improvements were observed in both the emotional (*Z* = −2.76, *p* = 0.007, *r* = 0.87) and Social WellBeing (*Z* = −2.69, *p* = 0.006, *r* = 0.85) subscales of the MHC-SF, indicating that the intervention was associated with positive changes in affective experiences and perceived social connectedness. This pattern is consistent with prior research identifying shared musical experiences as capable of strengthening social bonds and promoting positive emotional states through collective engagement and interpersonal synchrony (Tarr et al., 2014; Fancourt and Finn, 2019). No statistically significant change was observed in the psychological wellbeing subscale (*Z* = −1.94, *p* = 0.051, *r* = 0.61). This is consistent with the understanding that dimensions such as personal meaning, self-efficacy, and goal orientation develop through sustained engagement over longer periods and are unlikely to shift detectably within a 5-week period (Ryff, 1989). This finding does not diminish the significance of the improvements observed in other wellbeing dimensions; rather, it reflects the multidimensional nature of mental health and the time-dependent character of deeper psychological change.

### 4.4 Limitations and directions for future research

This study has several limitations that should be considered when interpreting the findings. First, the study employed a small-scale, single-group pretest–posttest pilot design with a limited sample size (*n* = 10), which restricts the generalisability of the results and does not allow for causal inferences. The absence of a control group means that observed changes cannot be attributed exclusively to the music-supported intervention. Alternative explanations, such as time effects, novelty effects, group interaction, or general adaptation processes, may also have contributed to the outcomes. Furthermore, although the observed effect sizes were large (*r* = 0.77–0.87), these estimates should be interpreted with caution given the small sample size, as effect size estimates derived from small samples may be unstable and may not accurately reflect population-level effects.

Second, the study relied on self-report measures to assess sociocultural adaptation, mental wellbeing, and mental health. While validated scales such as the SCAS-R, WHO-5, and MHC-SF were used, self-reported data may be influenced by subjective perceptions or social desirability bias. Furthermore, although established and widely validated instruments were used, internal consistency coefficients were not calculated for the present sample. Although alpha coefficients can technically be computed regardless of sample size, reliability estimates derived from very small samples (*n* = 10) are generally considered unstable and potentially misleading; therefore, their interpretation in the present context would be of limited value. This limitation is inherent to the pilot nature of the study, as the small sample size reflected the practical constraints of recruiting participants who could commit to attending all 5 weekly in-person sessions. Future research with larger samples should report internal consistency estimates for all scales used. Future research could strengthen the validity of the findings by incorporating researcher journals and behavioral observations.

Third, the journal data collected in this study were not subjected to formal qualitative analysis. The absence of a systematic coding procedure or thematic analysis means that the journal entries serve only as illustrative supplementary data, and no qualitative conclusions can be drawn from them. Future research would benefit from incorporating a rigorous qualitative or mixed-method strand including structured follow-up interviews or thematic analysis of journal data to more deeply explore participants’ experiences and, critically, to examine the specific role of music as distinct from other elements of the intervention.

Fourth, the short duration of the intervention represents an additional limitation. The program lasted for 5 weeks, which may not have been sufficient to produce substantial changes in certain dimensions such as academic adjustment, community involvement, or psychological wellbeing. Longitudinal research designs or extended intervention programs may provide a more comprehensive understanding of how these activities may influence adaptation and wellbeing over time. Furthermore, the study focused on a specific group of international students within a particular higher education context. Sociocultural adaptation experiences may vary depending on institutional environments and cultural backgrounds. Therefore, future research should aim to replicate and extend the present findings across different universities, countries, and cultural contexts to examine the broader applicability of music-supported cultural adaptation interventions.

Finally, future research could explore the effectiveness of other arts-based or culturally embedded approaches, such as theatre, dance, or visual arts. Investigating how other arts-based approaches may contribute to sociocultural adaptation and psychological wellbeing may support the development of more comprehensive and inclusive support strategies for international students in higher education. Future research employing larger and more diverse samples, controlled designs, and more comprehensive fidelity and psychometric reporting is recommended to build on these preliminary findings and strengthen the evidence base regarding the effectiveness of music-supported cultural adaptation interventions. In particular, the inclusion of an active control group such as a structured social activity group without music would allow for a more rigorous evaluation of the music-specific contribution to the observed outcomes.

## Data Availability

The original contributions presented in the study are included in the article/Supplementary material, further inquiries can be directed to the corresponding author.
