# Supplementary material for: Exploring the effects of music-supported cultural adaptation activities on international students’ sociocultural adaptation and wellbeing: a pilot study
Source: Front Psychol. 2026 Jun 5;17:1828413. doi: 10.3389/fpsyg.2026.1828413 (PMC13279408; doi:10.3389/fpsyg.2026.1828413)
Supplement: Supplementary file 1 [file Supplementary_file_1.DOCX]

**Appendix A**: Ethics Committee Approval

**Appendix B**: Demographic Information Form
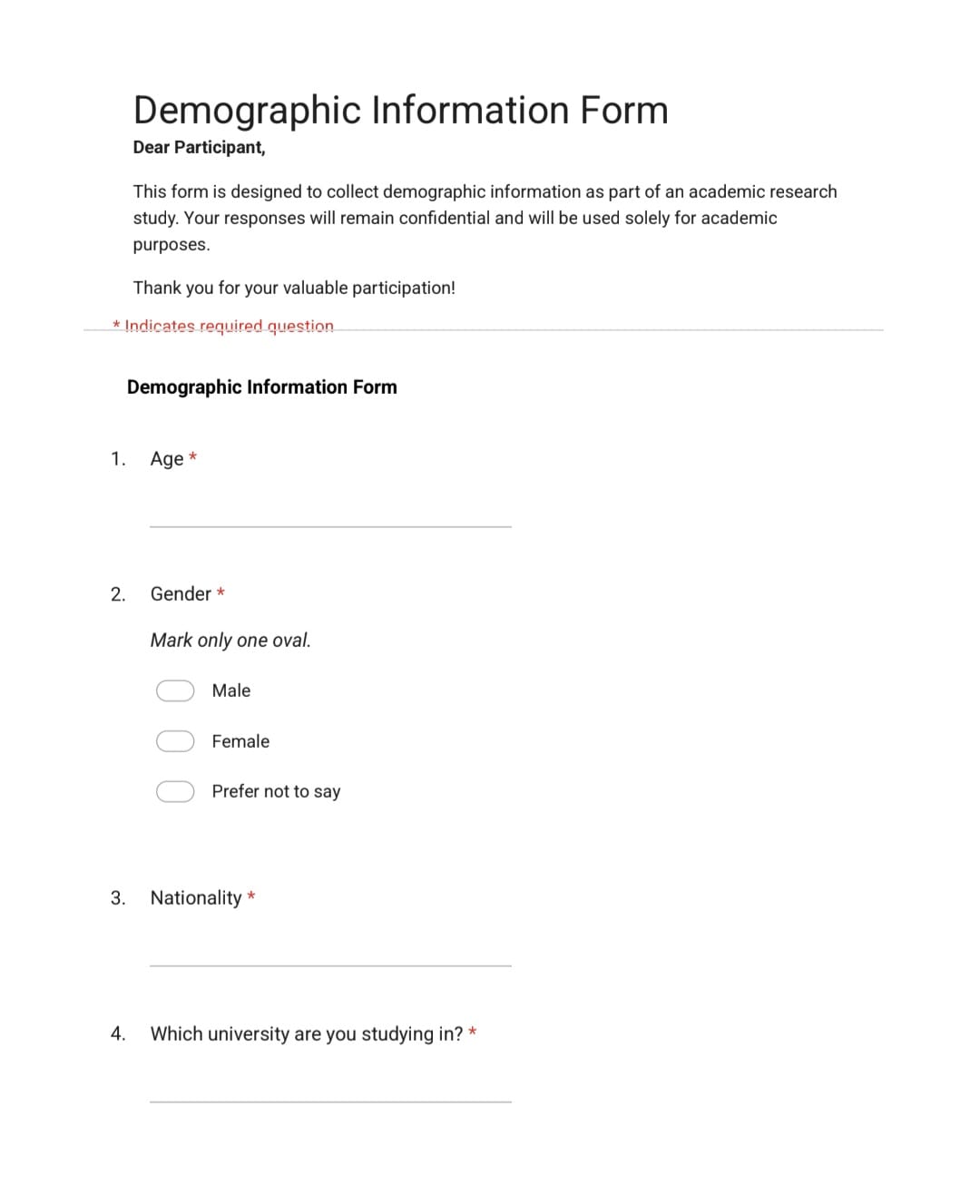


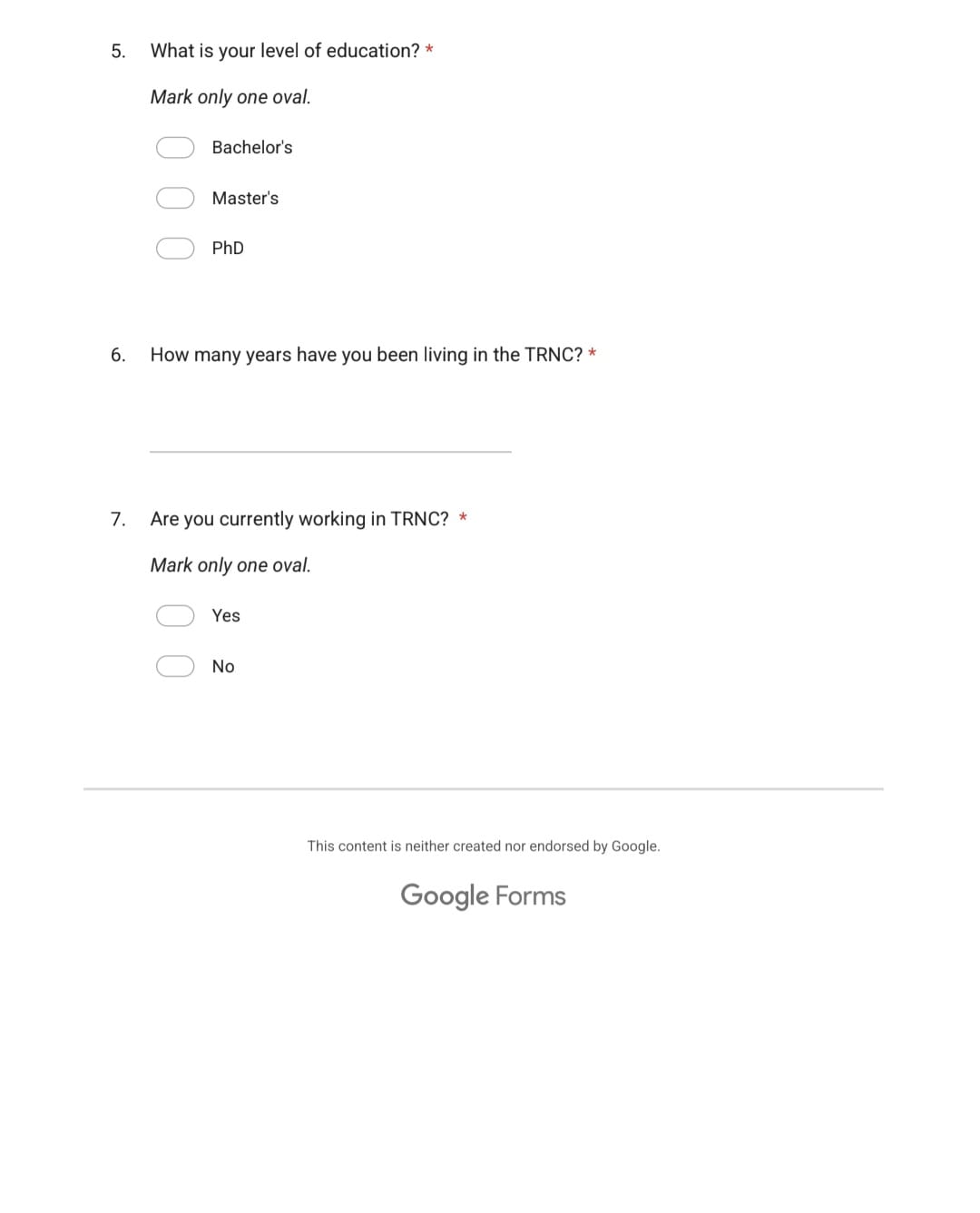


**Appendix C**: Participant Journal Guiding Questions

- How did you feel during today’s activities?
- Did the activities help you communicate or interact with other participants? Please explain.
- Did you learn something new about the local culture or environment? If yes, what did you notice?
- Did the activities influence your sense of connection with other people in the group?
- Did the session affect your mood, motivation, or general well-being in any way?
- Did the activities make you feel more comfortable participating in daily life?
- Is there any other experience or observation you would like to share about today’s activities?
